# Supplementary material for: Refinement of the Sugar Puckering Torsion Potential in the AMBER DNA Force Field
Source: J Chem Theory Comput. 2025 Jan 3;21(2):833–46. doi: 10.1021/acs.jctc.4c01100 (PMC11780733; doi:10.1021/acs.jctc.4c01100)
Supplement: Supplementary file 1 — ct4c01100_si_001.pdf [file ct4c01100_si_001.pdf]

Supporting Information:

Refinement of the Sugar Puckering Torsion Potential in the AMBER DNA Force Field.

Marie Zgarbová[1)], Jiří Šponer[2)], Petr Jurečka[1),*]

[1)] Department of Physical Chemistry, Faculty of Science, Palacky University, 17. listopadu 12, 77146 Olomouc, Czech Republic
[2)] Institute of Biophysics of the Czech Academy of Sciences, Kralovopolska 135, Brno 612 65, Czech Republic

Figure S1. QM and MM scans for the pseudorotation angle P.

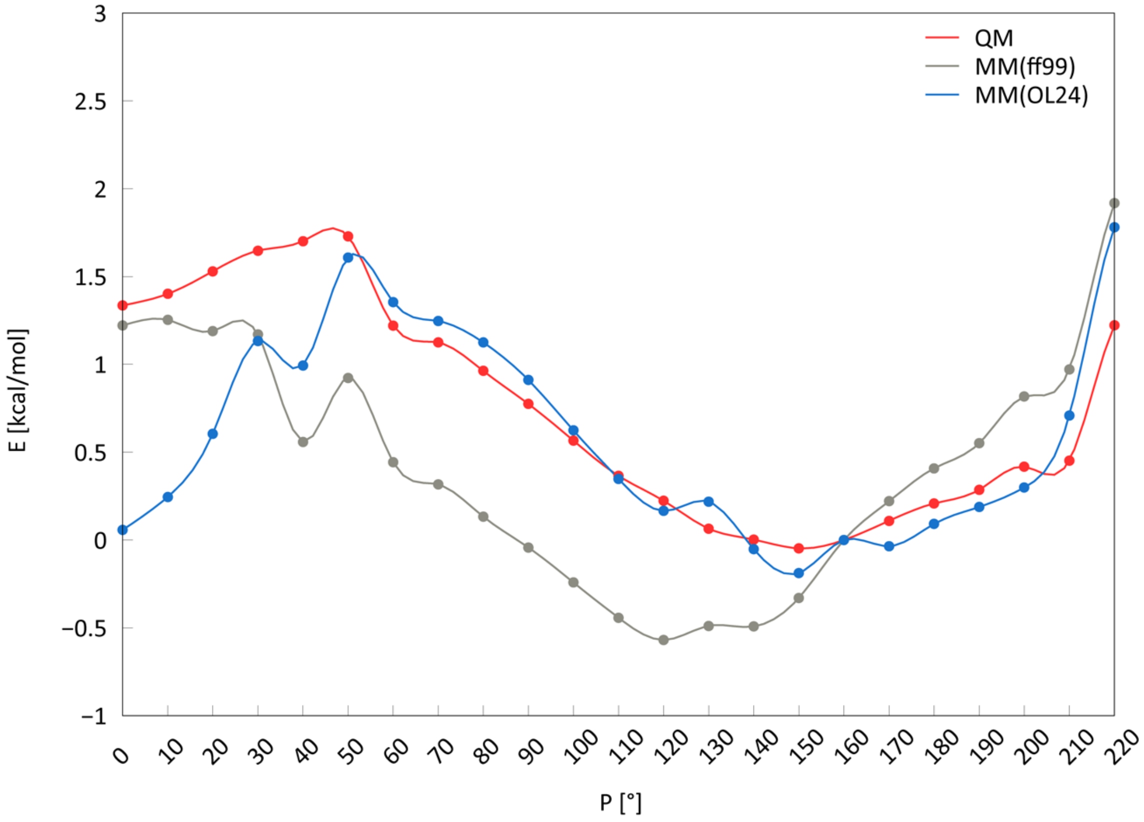

Figure S2A. Time series of sugar pucker in the DDD-r duplex simulation with OL24 parameters and SPC/E water model. One base pair at each end was excluded.

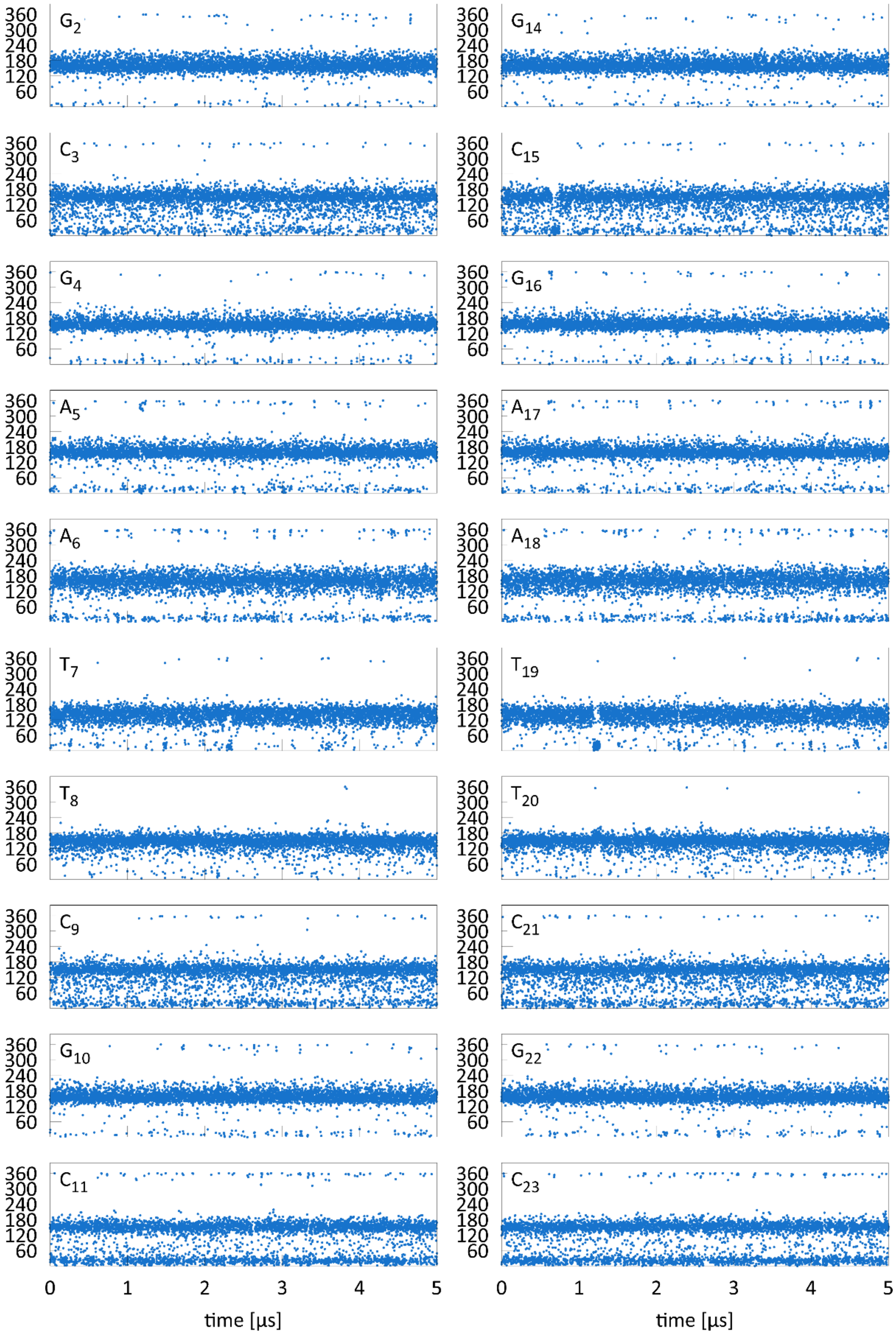

Figure S2B. Time series of sugar pucker in the DDD-r duplex simulation with OL24 parameters and TIP3P water model. One base pair at each end was excluded.

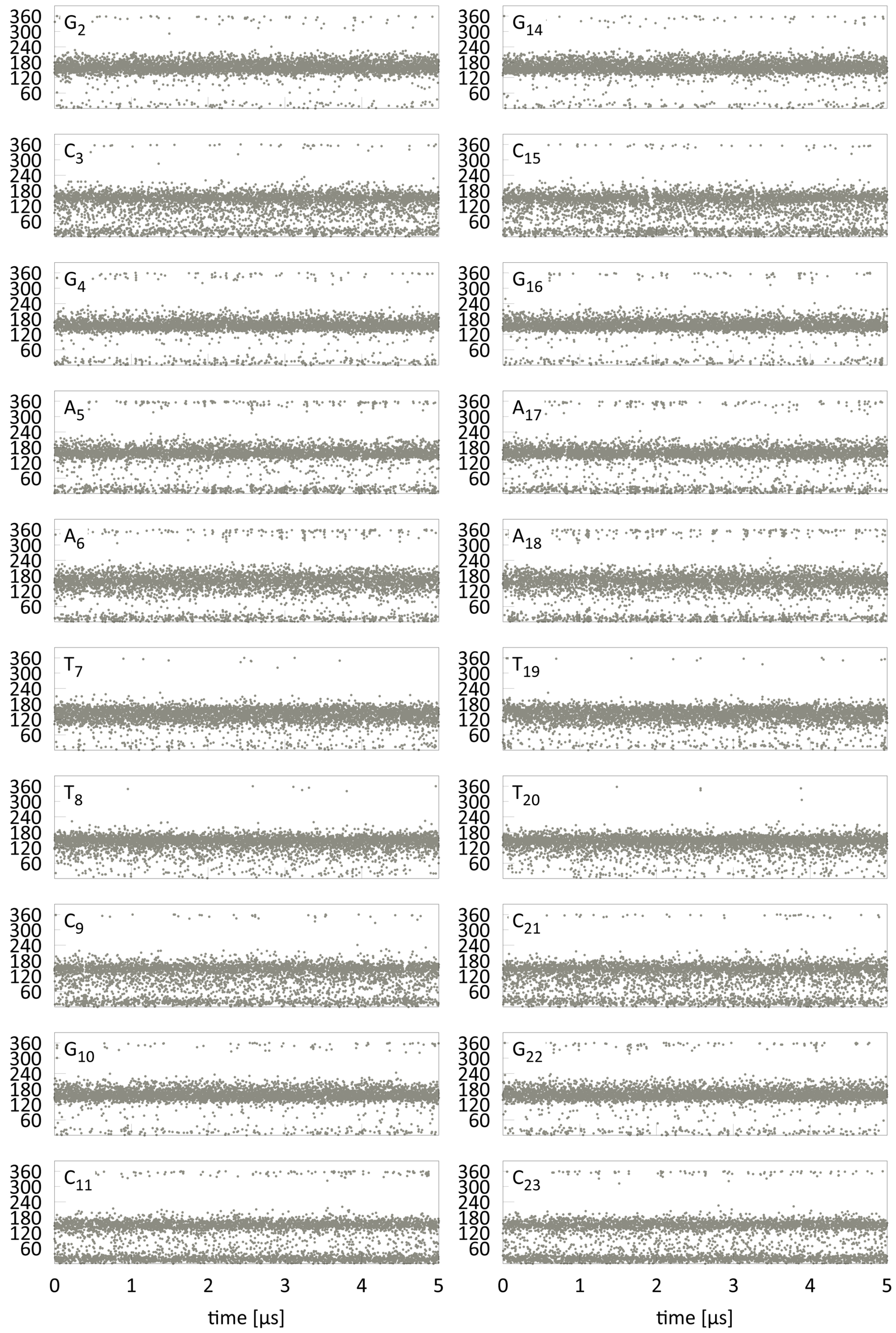

Figure S3. RMSD of simulated DDD relative to the 1BNA structure. Two base pairs at each end were excluded. The inset compares 5 μs OL24 simulations (SPC/E and TIP3P) with 2 μs OL21 simulation.

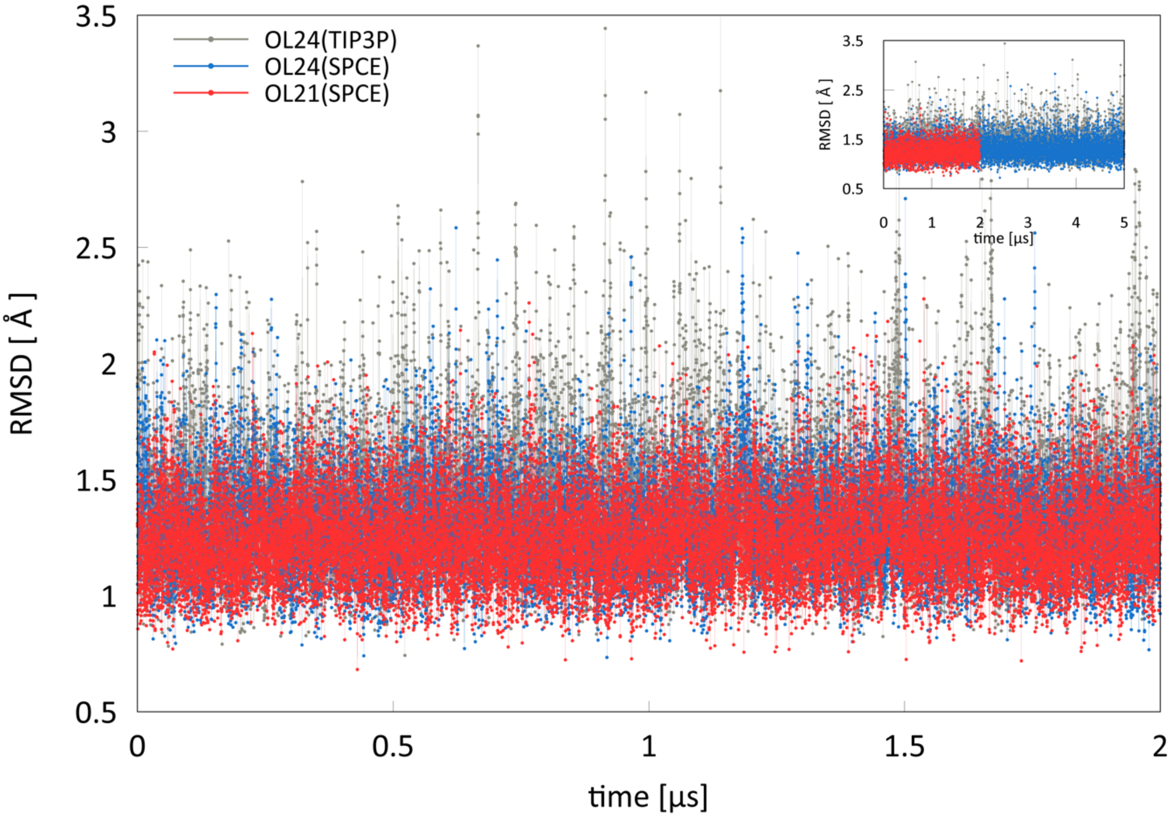

Figure S4. Fraying in unrestrained DDD dodecamer simulation: RMSD of the terminal bases C_1,G_{24} and G_{12},C_{13}.

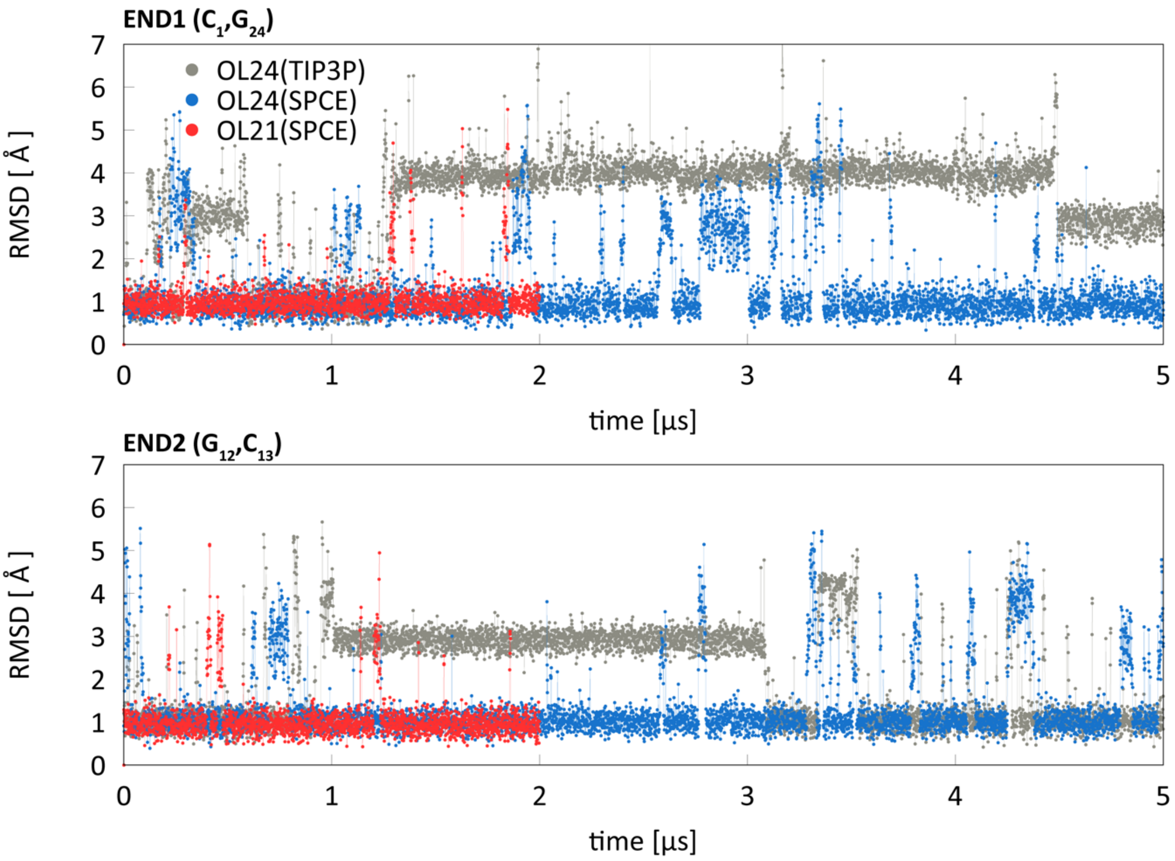

Figure S5. Sequence dependence of helical parameters in DDD-r simulations for OL21 and OL24 ffs.

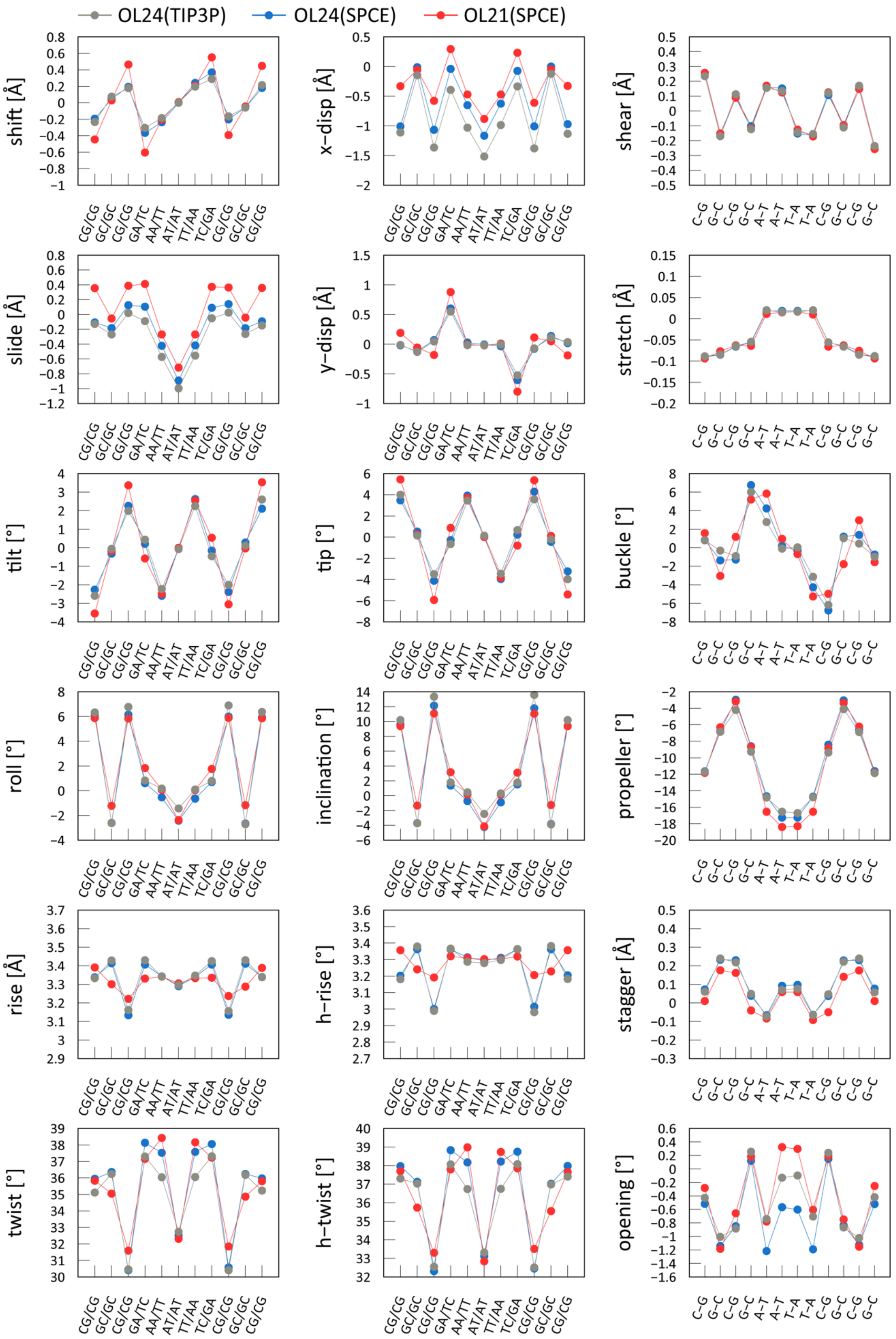

Figure S6. Dihedral angle distributions for DDD-r simulations (one base pair at each end was excluded) compared with distributions in non-complexed B-DNA and A-DNA X-ray structures (normalized to combined A+B data), and averages over five well-resolved DDD X-ray structures (see text).

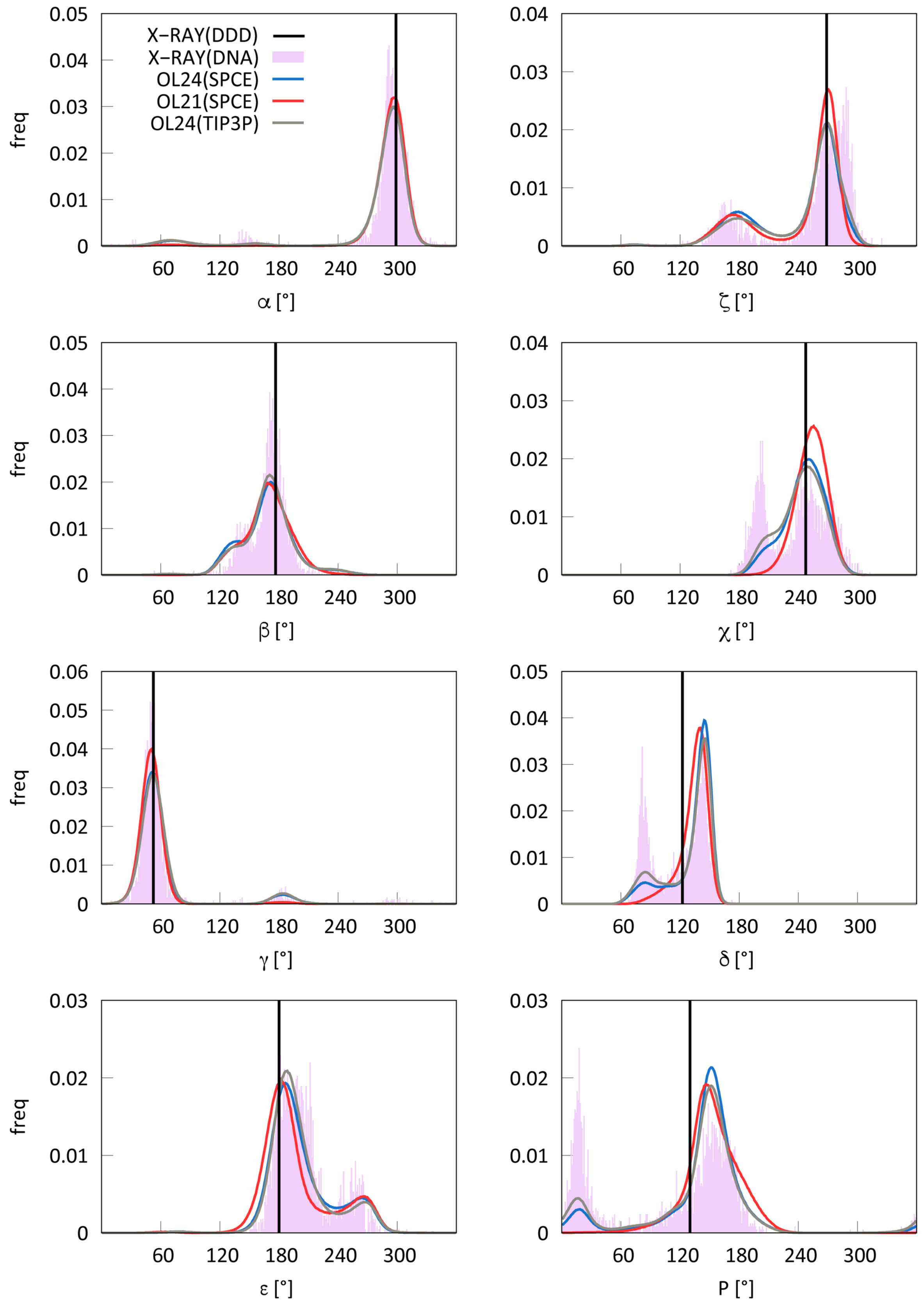

Figure S7. Helical and base pair parameter distributions and groove widths for DDD-r simulations (one base pair at each end was excluded) compared with distributions in non-complexed B-DNA and A-DNA X-ray structures, and averages over five well-resolved DDD X-ray structures (see text).

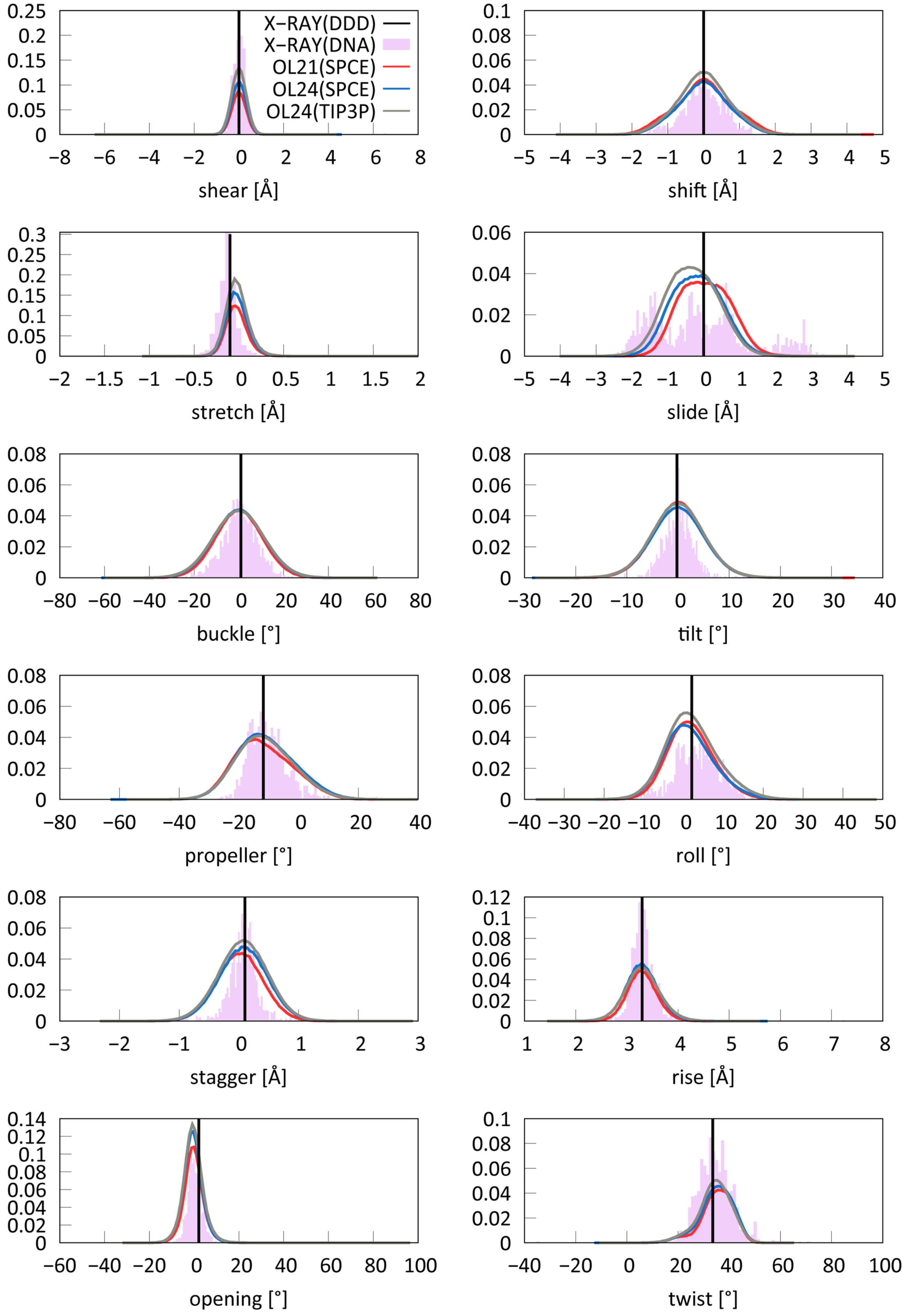

Figure S7. Continued

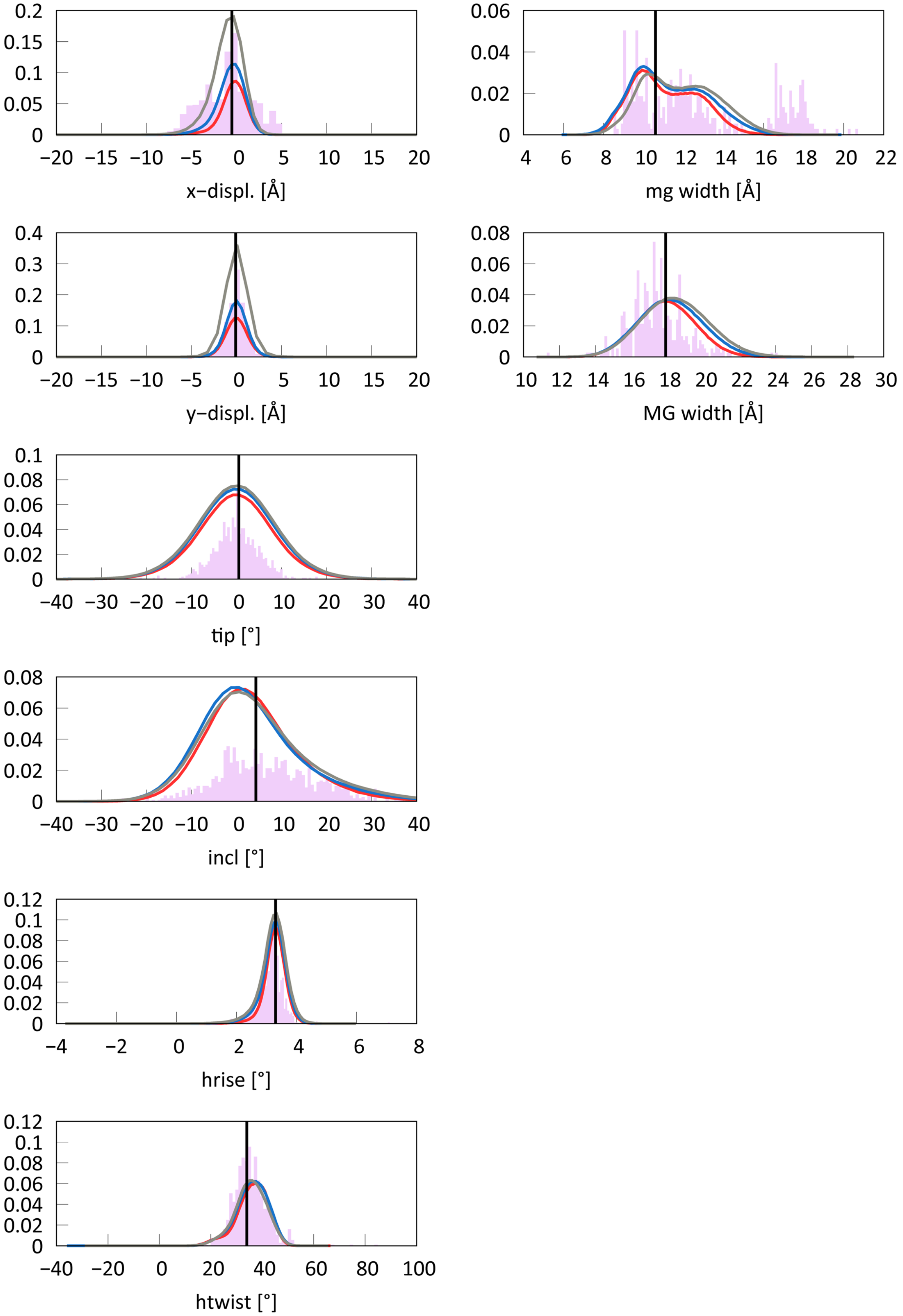

Figure S8. Three independent OL24 simulations of DDD starting from B-DNA conformation in 85% ethanol solution for two alternative water/ethanol parameterization variants. Reference values for inclination and x-displacement of A- and B-DNA forms are the same as in Table 4 in the main text. RMSD is relative to the idealized A-form.

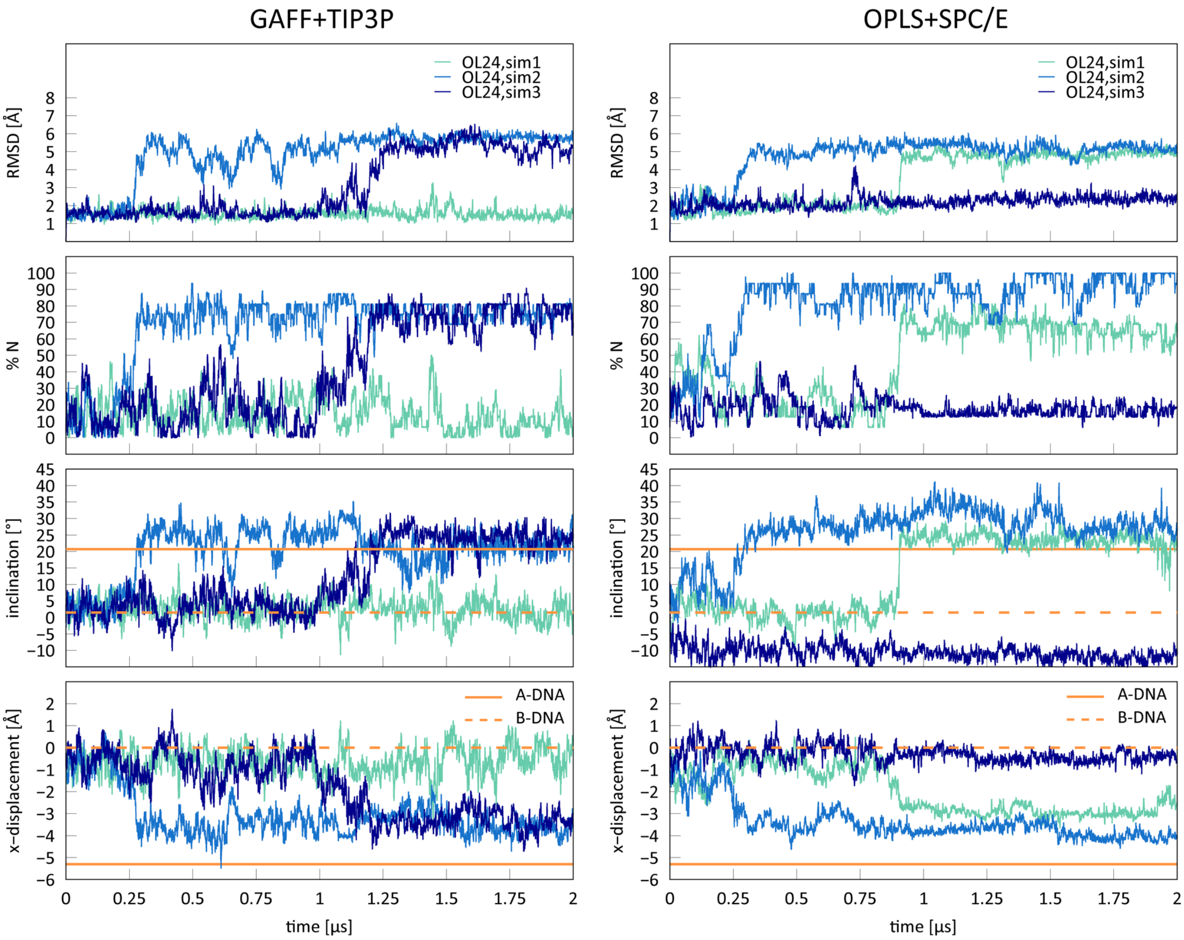

Table S1. OL24 deoxyribose dihedral angle parameters. Only the specified dihedral parameters were modified, while the remaining contributions to the δ and $\tau1$ torsions were retained from the original force field.

Torsion	n	$V_n/2$ [kcal/mol]	γ [°]
δ C5'-C4'-C3'-O3'	1	0.024	175.8
	2	4.413	86.83
	3	6.539	32.49
	4	4.132	1.235
	5	1.716	352.8
	6	0.801	0.902
$\tau1$ O4'-C1'-C2'-C3'	1	2.408	130.0
	2	7.000	192.5
	3	0.006	58.18
	4	3.343	320.3
	5	6.965	74.12
	6	4.111	250.3
